# Dynamic functional thalamocortical dysconnectivity in schizophrenia correlates to antipsychotics response

**DOI:** 10.1038/s41537-023-00371-y

**Published:** 2023-07-04

**Authors:** Mi Yang, Liju Liu, Hongmei Cui, Chijun Deng, Weisen Xiong, Guocheng Zhao, Shulin Du, Thomas R. Kosten, Huafu Chen, Zezhi Li, Xiangyang Zhang

**Affiliations:** 1 The fourth people’s hospital of Chengdu, Chengdu, China; 2 MOE Key Laboratory for Neuroinformation, High-Field Magnetic Resonance Brain Imaging Key Laboratory of Sichuan Province, University of Electronic Science and Technology of China, Chengdu, China; 3 Qingdao Mental Health Center, Qingdao University, Qingdao, China; 4 Department of Psychiatry, The Affiliated Brain Hospital of Guangzhou Medical University, Guangzhou, China; 5 Department of Psychiatry, Baylor College of Medicine, Houston, TX USA; 6 Epidemiology and Behavioral Science, MD Anderson Cancer Center, Houston, TX USA; 7 Sichuan Provincial Center for Mental Health, The Center of Psychosomatic Medicine of Sichuan Provincial People’s Hospital, University of Electronic Science and Technology of China, Chengdu, China; 8 Department of Psychology, University of Chinese Academy of Sciences, Beijing, China

**Keywords:** Schizophrenia, Schizophrenia, Biomarkers

## Abstract

Although many studies have showed abnormal thalamocortical networks in patients with schizophrenia (SCZ), the dynamic functional thalamocortical connectivity of individuals with SCZ and the effect of antipsychotics on this connectivity have not been investigated. Drug-naïve first-episode individuals with SCZ and healthy controls were recruited. Patients were treated with risperidone for 12 weeks. Resting-state functional magnetic resonance imaging was acquired at baseline and week 12. We identified six functional thalamic subdivisions. The sliding window strategy was used to determine the dynamic functional connectivity (dFC) of each functional thalamic subdivision. Individuals with SCZ displayed decreased or increased dFC variance in different thalamic subdivisions. The baseline dFC between ventral posterior-lateral (VPL) portions and right dorsolateral superior frontal gyrus (rdSFG) correlated with psychotic symptoms. The dFC variance between VPL and right medial orbital superior frontal gyrus (rmoSFG) or rdSFG decreased after 12-week risperidone treatment. The decreased dFC variance between VPL and rmoSFG correlated with the reduction of PANSS scores. Interestingly, the dFC between VPL and rmoSFG or rdSFG decreased in responders. The dFC variance change of VPL and the averaged whole brain signal correlated with the risperidone efficacy. Our study demonstrates abnormal variability in thalamocortical dFC may be implicated in psychopathological symptoms and risperidone response in individuals with schizophrenia, suggesting that thalamocortical dFC variance may be correlated to the efficacy of antipsychotic treatment.

**Registration**: ClinicalTrials.gov Identifier: NCT00435370. https://www.clinicaltrials.gov/ct2/show/NCT00435370?term=NCT00435370&draw=2&rank=1

## Background

Schizophrenia (SCZ) is one of the most severe psychiatric disorders, characterized by the disturbance of perception, thinking, emotion, cognition and behavior^1^. Over the past decade, accumulating evidence has shown the disruptions of the coordination of distributed brain networks in individuals with schizophrenia, which were commonly determined by detecting low-frequency blood oxygen level-dependent (BOLD) signal through resting-state functional magnetic resonance imaging (rs-fMRI) method^2,3^.

Recently, postmortem and neuroimaging studies have provided some direct evidence that thalamus is involved in SCZ^4–6^. The thalamus is the main relay station for multiple brain pathways involved in higher-order information processing and coordination^7^. Diffusion-weighted-imaging-based cortical fiber pathways suggested different thalamus substructures project to different cortical regions^8,9^; thalamocortical networks are topographically organized into parallel pathways, connecting distinct cortical areas to specific thalamic nuclei^10^. For example, the prefrontal cortex connected to the thalamus’s anterior and dorsal medial regions, while the sensorimotor cortex connected to the ventral and posterolateral regions of the thalamus^11^.

rs-fMRI can detect the correlation of transcerebral blood oxygen level based on the inherent low-frequency fluctuation of BOLD signal, avoiding many limitations of traditional task-based fMRI such as age, neuronal dysfunction, poor cooperation, or one-time and task-dependent response, and been proved useful for mapping brain networks^12^. rs-fMRI has recently demonstrated that the abnormalities of thalamic-cortical circuitry was related to the pathophysiology of SCZ^13,14^. Most previous fMRI studies used the whole thalamus as a region of interest (ROI) to investigate the functional characteristics in individuals with SCZ^15^. However, due to the complex function of the thalamus, it is difficult to determine whether the changes in thalamic-cortical functional connectivity (FC) in individuals with SCZ are caused by specific regions or the whole thalamus^16^. Fortunately, a connectivity-based parcellation method makes it possible to segment brain regions. Specifically, parceling the cortex into ROI corresponds to the subdivisions of the thalamic prefrontal lobe. Taking these ROI as seeds for FC analysis, the activity of each cortical region is related to specific regions of the thalamus^17–19^.

Recently, several studies have focused on the FC of thalamic subdivisions, providing more information about alterations in the thalamus-cortex coupling in individuals with SCZ. For example, Woodward et al. demonstrated lower prefrontal-thalamic connectivity and higher sensorimotor-thalamic connectivity^20^. They also showed the thalamocortical dysconnectivity in both chronic and early stages of psychosis including SCZ and bipolar I disorder^21^. Gong et al. demonstrated the loss of several thalamocortical networks in individuals with SCZ, and the change in connectivity was negatively correlated with severity of symptoms and course of disease^22^. However, the underlying mechanism of thalamocortical connectivity in the pathogenesis of SCZ remains unclear. Furthermore, few studies focus on the correlation between the changes of thalamocortical networks and antipsychotics efficacy.

Recent developments in dynamic functional connectivity (dFC) have revealed that human brain activity is time varying^23^. However, most connectivity analysis in SCZ focus on static functional connectivity, largely neglecting brain activity dynamics that have been reported to provide deeper insight into the underlying mechanisms of brain functions^24^. The dynamic analysis of the group difference in functional network connectivity can obtain more information about abnormal connection modes, which cannot be observed in static analysis^23,25^.

Therefore, to obtain a more precise diagnosis and prognosis of schizophrenia, we conducted a prospective study in drug-naive first-episode (DNFE) individuals with SCZ to investigate the effect of risperidone on the abnormal patterns of dFC of thalamus subdivisions.

## Materials and methods

### Participants

This study protocol was approved by Institutional Review Board of Beijing Hui-Long-Guan hospital (No. SCH-A01). This was a post-hoc study from our previous study (ClinicalTrials.gov Identifier: NCT00435370). Written informed consents were obtained from all patients and healthy controls. 43 DNFE individuals with SCZ who met the DSM-IV diagnostic criteria for SCZ were enrolled, and of whom were ill for <5 years. We recruited 29 healthy control subjects without any family or personal history of mental disease through local community advertisements. All participants underwent physical examinations and laboratory tests, and provided a complete medical history. Any participant with severe medical conditions, illegal drug or alcohol abuse/dependence was excluded. As shown in Table 1, the two groups showed no statistical differences in age, sex, education, BMI and smoking (all *p* > 0.05).

**Table 1:** Demographical and clinical characteristics of the schizophrenia group and healthy control group (mean ± SD).

Variable	SZ (37)	HC(27)	χ^2^/Fisher/F	*p*
**Age (y)**	28.81 ± 10.63	27.37 ± 7.84	0.36	0.55
**Sex (N, %)**				
Male	18 (48.6%)	11 (40.7%)	0.39	0.53
Female	19 (51.4%)	16 (59.3%)		
**Education (y)**	12.30 ± 3.29	12.00 ± 3.84	0.13	0.72
**BMI (kg/m**^**2**^**)**	21.21 ± 3.19	22.43 ± 3.64	2.36	0.13
**Smoking (N, %)**	4 (10.8%)	1 (3.7%)	1.10	0.39
**Onset age (y)**	26.11 ± 9.92			
**PANSS total score**	93.05 ± 25.50			
P subscore	25.95 ± 6.85			
N subscore	21.22 ± 9.87			
G subscore	45.30 ± 14.41			

### Clinical assessments and prospective cohort study

All participants were screened by two separate psychiatrists using the Structured Clinical Interview for DSM-IV (SCID)^26^. We assessed psychotic symptoms by the positive and negative syndrome scale (PANSS)^27,28^. PANSS subscales include PANSS positive subscale score, PANSS negative subscale score, PANSS general psychopathology subscale score. In this study, all the raters have been trained together on the assessment of PANSS before the project start. Then, all the raters assessed one patient simultaneously, and finally the inter-rater correlation coefficients were obtained and were >0.8. The reduction rate of PANSS total score was calculated by the formula (PANSS_*baseline*_ − PANSS_*12th week*_)/(PANSS_*baseline*_ − 30) × 100%^29^. We identified patients as responders, if they showed at least 50% reduction rate on their PANSS total scores by week 12^30^.

At baseline, all participants underwent MRI scans; six were excluded for poor MRI data. Only 20 completed their MRI scans at the end of week 12, and three had great head movements, leaving 17 patients with valid brain imaging data at week 12. We obtained the fMRI data on patients before they started any medications.

Among the 29 healthy controls, two were excluded for significant head movement, and leaving 27 for the image analysis.

The patient each received a stable dose of risperidone and was followed up for 12 weeks after their fMRI scans. The risperidone titration started at 1 mg/d and increased to 3–6 mg/d in the first week, and these doses remained stable for the next 11 weeks. We allowed chloral hydrate or lorazepam to treat short term insomnia, and benzazol Hydrochloride to treat extrapyramidal symptoms. No other antipsychotics or antidepressants were allowed during this study.

### Imaging acquisition and preprocessing

rs-fMRI data were acquired on a 3.0 Tesla scanner (General Electric) equipped with an 8-channel brain-phased array coil. Functional data were obtained using a gradient-echo echo-planar imaging (EPI) sequence (TR/TE = 2000ms/30 ms, flip angle = 90°, slice sickness = 4 mm, slice number = 33, field of view = 240 × 240 mm^2^, matrix size = 64 × 64, voxel size = 3.75 × 3.75 × 4 mm^3^, 190 time points collected).

Image preprocessing used the Data Processing Assistant for Resting-State fMRI (DPARSF, V4.3) (http://rfmri.org/DPARSF). The steps are as follows: We excluded the first five brain volumes, correction of slice timing and realignment. Subsequent images were normalized to the Montreal Neurologic Institute (MNI) space and spatial resampling conducted to make each voxel size is 3 × 3 × 3 mm^3^. To produce a high signal-to-noise ratio, the image data was smoothed with a full width at half maximum (FWHM) of 8 mm Gaussian kernel. We used multiple linear regression derived from Volterra expansion regression analysis for statistical adjustments to some false variance sources such as head motion parameters and averaging of signals from white matter and cerebrospinal fluid. The linear trend of the functional image was then eliminated by detrending and time bandpass filtering (0.01–0.10 Hz).

### Definition of thalamus subdivisions by “Winner-take-all” strategy

We used the “Winner take all” strategy to segment the thalamus^17,18,31^, after defining six cerebral cortical regions (frontal cortex, motor cortex, temporal cortex, sensory cortex, posterior parietal cortex, occipital cortex) based on The Harvard—Oxford template^32^. We then extracted the average BOLD time course from each of these six cortical regions. We then extracted the thalamic template from the automatic structure marker template. For each voxel in the thalamus of each subject, we calculated the partial correlations of the voxel’s BOLD signal with the six cerebral cortical regions’ BOLD signal. We then compared the sizes of these six partial correlation coefficients, and assigned each thalamic voxel to one cerebral cortex subregion by "Winner-take-all" strategy, which is to mark the voxel based on the maximum value of the six partial correlations.

### dFC analysis

We used the sliding window method from the dynamic BC toolbox (www.restfmri.net/forum/DynamicBC) to obtain the whole brain dFC map of each thalamic subregion and generated 28 windows with the window size set to 50 TRs and the step size to 5 TRs^33,34^. We obtained Pearson’s correlations between the average time series between the six thalamus subregions and each other voxel in each window, and used Fisher’s Z transform to convert the correlation coefficients into z scores to improve normality. Each participant got a set of sliding window correlation map with the standard deviation of the z-value at each voxel as a variance estimate for each participant’s dFC time series at each thalamic subregion.

For each of the six thalamic subregions, an independent t-test was conducted to compare dFC variances at baseline between patients and healthy controls, with adjustment for age, sex, education and head motion. We conducted multiple comparisons were corrected by following two step: (i) a Bonferroni correction with *P* < 0.05 was used to correct six thalamocortical comparisons, as a result of a correction threshold of *P* < 0.005 for each thalamocortical comparison; and (ii) AlphaSim was then conducted for each thalamocortical voxel-wise comparison (with a height threshold of *P* < 0.005 for all cortical areas, and extent thresholds for each cortical size) (http://www.restfmri.net).

### Statistical analysis

For continuous variables, Kolmogorov-Smirnov one-sample test was for normality test, and t-tests or analysis of variance (ANOVA) for group comparison, but chi-square test for category variables.

To examine the dFC variance between responders and non-responders, the repeated measures multivariate analysis of variance (RM-MANOVA) method was taken on each PANSS subscale score, with adjusting for confounding covariates. The multivariate omnibus test was for determining statistical significance. If only the group × time interaction had a significant effect, the group difference was assessed by ANOVA at week 12, adjusting for baseline dFC value, age, sex, education, BMI and illness duration. Bonferroni correction was used for multiple comparisons. PASW Statistics 23.0 (SPSS, Inc., Chicago) was applied for statistical analysis.

## Results

### Parcellation of the thalamus

The bilateral thalamus was parcellated into six nearly symmetrical subdivisions in Supplementary Figure 1. These subdivisions were designated as anterior and dorsomedial regions (AD), ventral lateral regions (VL), ventral posterior-lateral portions (VPL), posterior medial (PM), lateral areas (L) and lateral posterior nucleus (LP) of the thalamus.

### Baseline dFC variance of thalamus subdivisions between patients and healthy controls

**Fig. 1:**
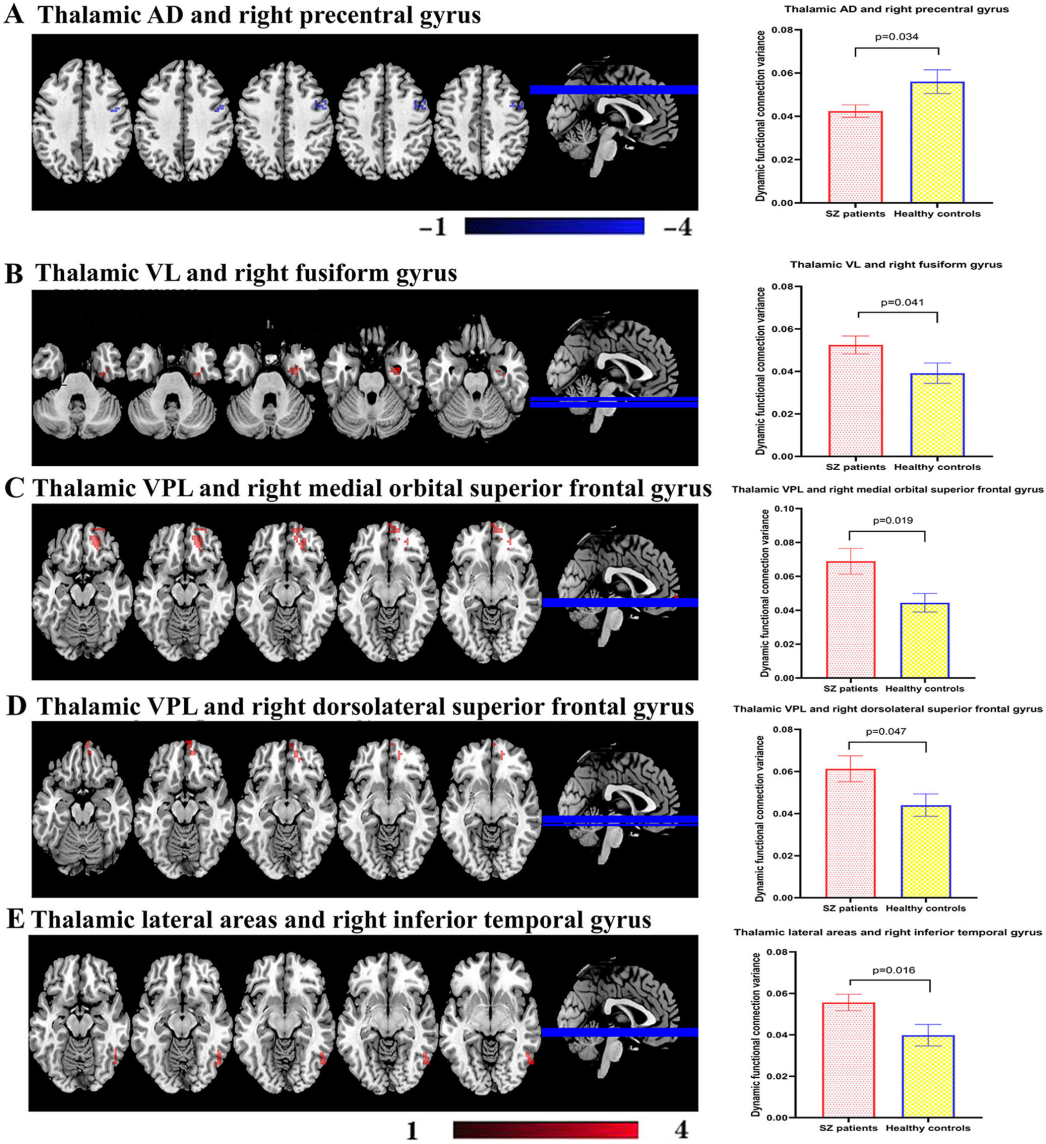
Differences in the baseline dynamic functional connectivity of thalamus subdivisions between patients and healthy controls. **A** Patients have significantly weakened dFC variances between thalamic AD and right precentral gyrus compared with controls (*p* = 0.034). **B**–**E** The strengthened dFC variances in thalamic VL to right fusiform gyrus (*p* = 0.041), thalamic VPL to right medial orbital superior frontal gyrus (*p* = 0.019), thalamic VPL to right dorsolateral superior frontal gyrus (*p* = 0.047) and thalamic lateral areas to right inferior temporal gyrus (*p* = 0.016). AD anterior and dorsomedial regions, dFC dynamic functional connectivity, VL ventral lateral regions, VPL ventral posteriorlateral portions.

Kolmogorov-Smirnov test showed the data was normally distributed. In Fig. 1, after correction for multiple comparisons, patients and healthy controls showed significant differences in dFC variance of thalamic AD and right precentral gyrus (p_Bonferroni_ = 0.034), thalamic VL and right fusiform gyrus (p_Bonferroni_ = 0.041), thalamic VPL and rmoSFG (p_Bonferroni_ = 0.019), thalamic VPL and rdSFG (p_Bonferroni_ = 0.047), thalamic lateral areas and right inferior temporal gyrus (p_Bonferroni_ = 0.016).

### Association between baseline dFC variance of thalamus subdivisions and clinical symptoms

**Fig. 2:**
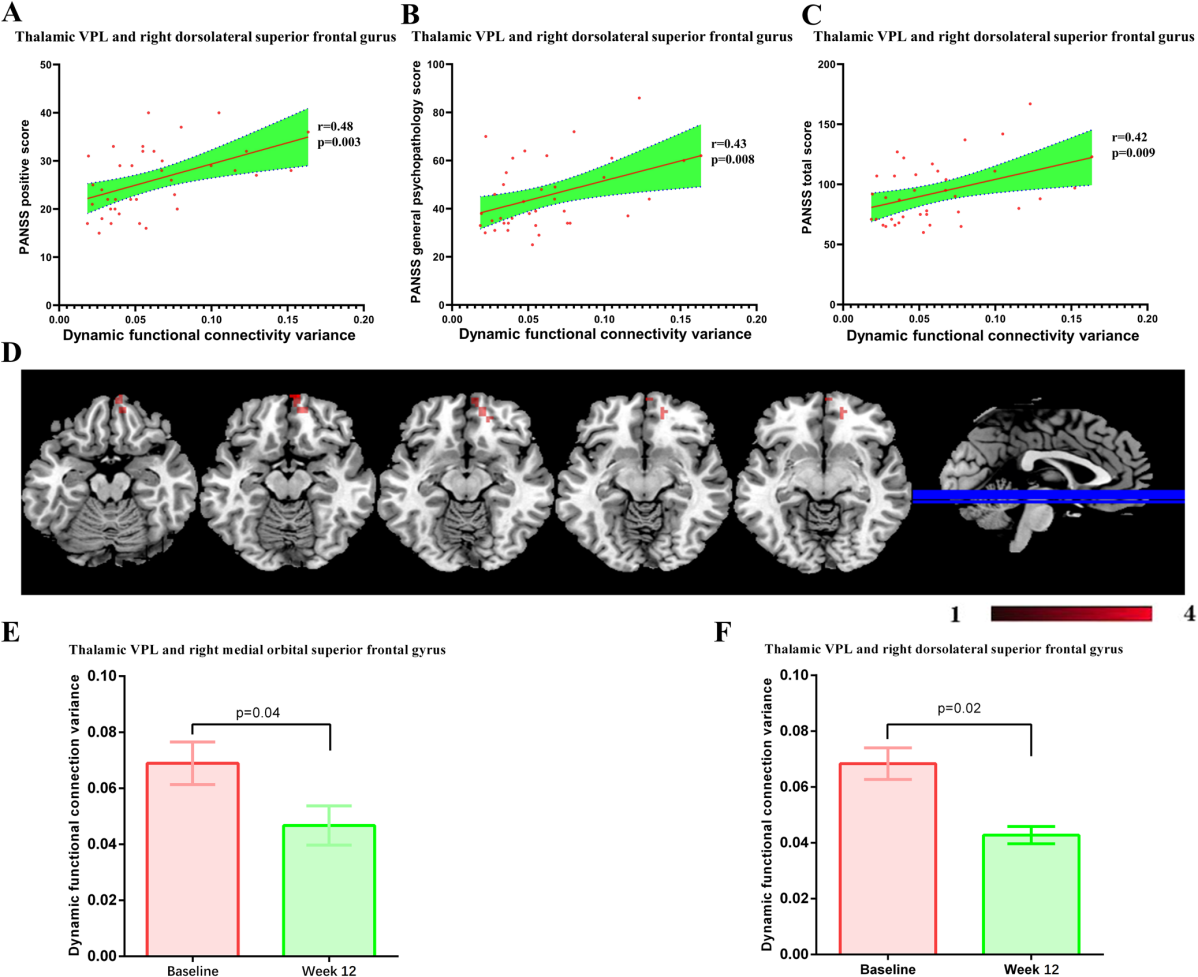
Effects of 12-week risperidone treatment on dynamic functional connectivity of thalamus subdivisions. **A**–**C** The baseline dFC variance of thalamic VPL and right dorsolateral superior frontal gyrus was associated with the PANSS positive subscale score (*r* = 0.48, *p* = 0.003), PANSS general psychopathology subscale score (*r* = 0.43, *p* = 0.008) and PANSS total score (*r* = 0.42, *p* = 0.009). VPL: ventral posterior-lateral portions. **D**–**F** Risperidone deceased the dFC between thalamic VPL and right medial orbital superior frontal gyrus (*p* = 0.04), and dFC between thalamic VPL right dorsolateral superior frontal gyrus (*p* = 0.02). VPL: Ventral posterior-lateral portions.

As shown in Fig. 2A–C, after correction for multiple comparisons, the baseline dFC variance of thalamic VPL and rdSFG was associated with the PANSS positive subscale score (*r* = 0.48, *p* = 0.003, p_Bonferroni_ = 0.01), PANSS general psychopathology subscale score (*r* = 0.43, *p* = 0.008, p_Bonferroni_ = 0.03) and PANSS total score (*r* = 0.42, *p* = 0.009, p_Bonferroni_ = 0.04).

### Effects of 12-week risperidone treatment on dFC variance between thalamus subdivisions and brain regions

After 12 weeks of treatment with risperidone, the patients displayed decreased PANSS total and all three subscale scores (all *p*_Bonferroni_ < 0.05). In Fig. 2D–F, after 12 weeks of risperidone treatment, the dFC variances between thalamic VPL and rmoSFG (*t* = 2.24, *p* = 0.04) or rdSFG (*t* = 2.66, p = 0.02) decreased significantly.

### Changes in dFC variance of thalamus subdivisions correlate to 12-week risperidone treatment efficacy

**Fig. 3:**
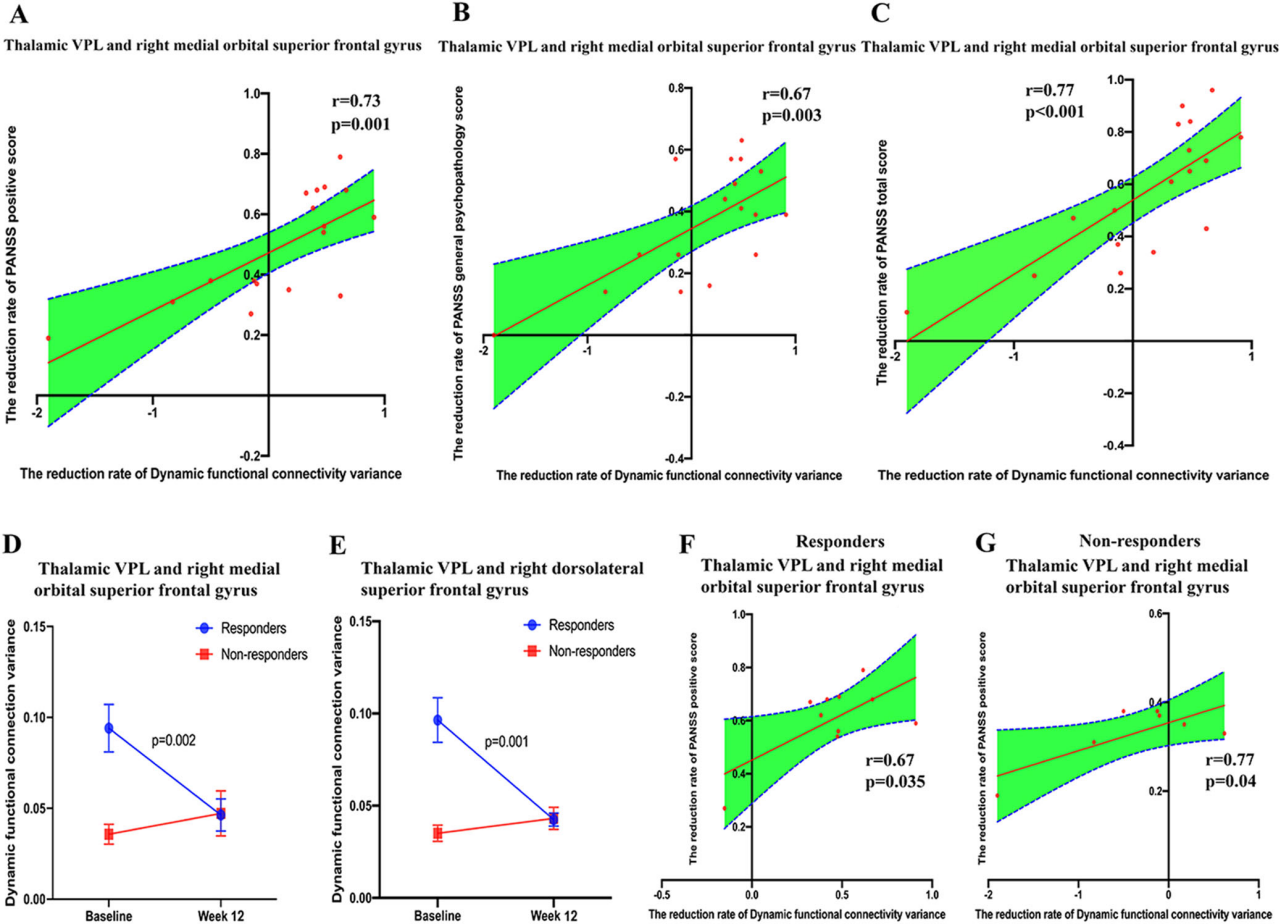
Dynamic functional connectivity of thalamus subdivisions correlates to 12-week risperidone treatment efficiency. **A**–**C** The reduction rate of dFC variance in thalamic VPL and right medial orbital superior frontal gyrus, was associated with the reduction rate of PANSS positive score (*r* = 0.73, *p* = 0.001), PANSS general psychopathology score (*r* = 0.67, *p* = 0.003) and PANSS total score (*r* = 0.77, *p* < 0.001). **D**, **E** The dFC variance of thalamic VPL with the right medial orbital superior frontal gyrus (*p* = 0.002) and the right dorsolateral superior frontal gyrus (*p* = 0.001) decreased in the responder group, but not in the non-responder group. **F**, **G** The reduction rate of dFC variance within the thalamic VPL (and right medial orbital superior frontal gyrus) was associated with the reduction rate of PANSS positive score both in the responder group (*r* = 0.67, *p* = 0.035) and in the non-responder group (*r* = 0.77, *p* = 0.04). VPL Ventral posterior-lateral portions.

In Fig. 3A–C, after 12 weeks of risperidone treatment, the reduction rate of dFC variance in thalamic VPL and rmoSFG was associated with the reduction rate of PANSS positive score (*r* = 0.73, *p* = 0.001, *p*_Bonferroni_ = 0.004), PANSS general psychopathology score (*r* = 0.67, *p* = 0.003, *p*_Bonferroni_ = 0.01) and PANSS total score (*r* = 0.77, *p* < 0.001, *p*_Bonferroni_ < 0.001).

The patients were divided into responder patients (*n* = 7) and non-responder patients (*n* = 10). RM-MANOVA showed a significant group × time effect of MRI indicators (Wilks’ lambda *F* = 12.585, *p* = 0.004), and on the variances of dFC between thalamic VPL and rmoSFG (Wilks’ lambda *F* = 11.59, *p* = 0.008) or rdSFG (Wilks’ lambda *F* = 11.13, *p* = 0.009). dFC variance of thalamic VPL with rmoSFG (*p* = 0.002) and rdSFG (*p* = 0.001) decreased significantly in the responder group as shown in Fig. 3D, E. Figure 3F, G showed the reduction rate of dFC variance within the thalamic VPL (rmoSFG) was associated with the reduction rate of PANSS positive score both in the responder group (*r* = 0.67, *p* = 0.008, p_Bonferroni_ = 0.03) and in the non-responder group (*r* = 0.77, *p* = 0.01, p_Bonferroni_ = 0.04).

## Discussion

This study had several main findings: (1) There were differences in dFC variance of cortical regions and the thalamus subdivisions between SCZ and healthy controls. (2) Baseline dFC variance between thalamic VPL and rdSFG was associated with the severity psychotic symptoms in individuals with SCZ. (3) the dFC variances between thalamic VPL and rmoSFG or rdSFG decreased significantly after 12 weeks of risperidone treatment. Furthermore, the reduction rate of the dFC between the thalamic VPL and rmoSFG was associated with the reduction rate of the PANSS score. Interestingly, the dFC variances between thalamic VPL and rmoSFG decreased significantly in the responder group, but not in the non-responder group. Furthermore, the change in dFC variances between the thalamic VPL and the averaged whole brain signal was associated with the efficacy of risperidone treatment.

Thalamus is a critical node in large-scale brain networks, and thalamocortical interactions are emerging as a key component of “dysconnectivity” in SCZ. Thalamocortical connectivity alterations in SCZ are bidirectional and regionally specific. For example, contrary to the general notion that neural connectivity is overall reduced in SCZ, previous studies have shown that individuals with SCZ have pathways of distinct intrinsic hypoconnectivity and hyperconnectivity from prefrontal-limbic and sensorimotor cortices to thalamic nuclei^35,36^. Our study showed that individuals with SCZ displayed differences in dFC variance of cortical regions and the thalamus subdivisions between SCZ and healthy controls. For example, Anticevic et al. found that individuals with SCZ exhibited lower connectivity between the thalamus and regions of prefrontal cortex, striatum, and cerebellum compared to healthy controls^3^. Woodward et al. reported that individuals with SCZ displayed decreased prefrontal-thalamic connectivity and increased motor/somatosensory-thalamic connectivity compared to healthy controls^20^. They also demonstrated the same results in both chronic and early-stage psychosis patients^21^. Tu et al. also reported that individuals with SCZ displayed a decreased cingulo-insular (CO) network, which comprises prefrontal cortex and thalamus, but increased sensorimotor networks compared to healthy controls^37^. Çetin et al. revealed increased thalamus-posterior temporal lobe connectivity in individuals with SCZ^38^. In addition, Anticevic et al. also showed decreased prefrontal-thalamic connectivity and increased sensorimotor-thalamic connectivity in 243 individuals at clinical high risk for psychosis^16^. Interestingly, Lui et al. found decreased prefrontal-thalamic connectivity in the first-degree relatives of individuals with SCZ^39^. These results suggest that cortico-thalamic abnormal connectivity may be an inherent feature of SCZ. Kaufmann et al. reported that individuals with SCZ had lower sensorimotor connectivity than healthy controls^40^. Recently, Gong et al. also showed reduced sensorimotor connectivity in individuals with SCZ^22^.

The possible reason of differences in dFC variances of cortical regions and the thalamus subdivisions between SCZ and healthy controls may be explained by a neurodevelopmental hypothesis of SCZ^20^. It is well established that thalamocortical FC appears differently at the stages of child, adolescent and adult^41^. Specifically, in typical brain development, prefrontal-thalamic connectivity in children and adolescents is sparse, and these connections develop during the transition from adolescence to adulthood. However, sensorimotor-thalamic connectivity seems to peak in adolescence and then decrease in adulthood^41^. In addition, contrary to prefrontal-thalamic connectivity, the temporal-thalamic connectivity decreases with age from child to adolescent to adult. Our results are contrary to the normal rule of brain development, since individuals with SCZ displayed decreased prefrontal-thalamic connectivity and increased sensorimotor-thalamus and temporal-thalamus connectivity. Our results followed the neurodevelopmental hypothesis of SCZ, by showing abnormal brain development from adolescence to adulthood, specifically showing impaired connectivity of prefrontal-thalamus, sensorimotor-thalamus and temporal-thalamus.

Our study found that the baseline dFC variances between the ventral posterior-lateral portions of the thalamus and the right Superior frontal dorsolateral gyrus was associated with the PANSS total score, the positive and general psychopathology subscores. The somatomotor networks damage in SCZ leads to abnormal perceptual processing (e.g., hallucinations), psychomotor disturbances and cognitive impairments^4,42,43^. Perceptual symptoms (e.g., hallucinations) are closely associated with positive and general psychopathology symptoms in individuals with SCZ consistent with our results. In addition, Anticevic et al. reported the associations between sensorimotor-thalamic hyperconnectivity and general psychopathology in individuals with SCZ^3^, and between the auditory-sensorimotor-thalamus hyperconnectivity and the PANSS positive symptoms subscore^44^.

Most importantly, this prospective cohort of DNFE individuals with SCZ demonstrated that 12 weeks of risperidone treatment decreased the dFC variance between the thalamic VPL and rmoSFG. Furthermore, the reduction in dFC dFC variance between the thalamic VPL and rmoSFG was associated with the reduction in PANSS scores. Interestingly, the dFC variance between the thalamic VPL with rmoSFG and with rdSFG decreased significantly in responders, but not in non-responders. The ventral posterolateral thalamus is regarded as the principal cerebellar thalamic relay nucleus, and is reciprocally connected with the primary motor area, the premotor area and the supplementary motor area^45^. Previous evidence reported that individuals with SCZ had reduced dopamine synthesis capacity in the sensorimotor striatum, which correlated positively with increased auditory-sensorimotor network connections with the ventrolateral-thalamus^44^. On the other hand, Ito et al. demonstrated a negative correlation between baseline dopamine synthesis capacity and risperidone induced changes in dopamine synthesis capacity, which they interpreted as risperidone stabilizing the dopamine synthesis capacity and dopaminergic neurotransmission response^46^.

The thalamus is closely related to the physiological mechanisms of SCZ, and a potentially promising method to link these neuroimaging biomarkers to potential neural circuit mechanisms uses the "computational psychiatry" approach. Since a key factor in thalamic cortical disconnection in SCZ is its regional specificity, it is important to consider the potential sources of inherent heterogeneity of cellular and circuit characteristics across cortical and thalamic structures.

There were several limitations in this study. First, although it is difficult to recruit DNFE patients, the sample size was moderate. Further studies with a larger sample size should be conducted to confirm our results. Second, although the application of risperidone monotherapy in this prospective cohort study eliminated the effects of different antipsychotics, further study should be conducted to investigate the effects of other antipsychotic agents on thalamocortical dysfunction.

This study demonstrates abnornmal dFC variance in individuals with SCZ. The dFC variances between the ventral posterior-lateral thalamus and whole brain were associated with the severity of psychotic symptoms and the efficiency of the antipsychotic risperidone.

## Supplementary information

Supplementary Fig. 1

## Data Availability

The data that support the findings of this study are available from the corresponding author X.Z. upon reasonable request.
