# Supplementary figures and images for: Dynamic functional thalamocortical dysconnectivity in schizophrenia correlates to antipsychotics response
Source: Schizophrenia (Heidelb). 2023 Jul 4;9(1):40. doi: 10.1038/s41537-023-00371-y (PMC10319786; doi:10.1038/s41537-023-00371-y)

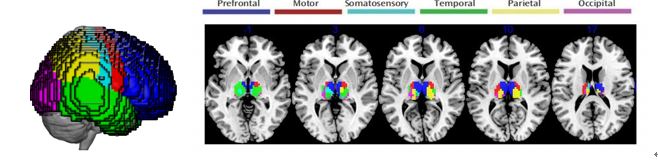

Supplement: Supplementary file 1 — Supplementary Fig. 1 [file 41537_2023_371_MOESM1_ESM.jpg]
